# PIN1P1 is activated by CREB1 and promotes gastric cancer progression via interacting with YBX1 and upregulating PIN1

**DOI:** 10.1111/jcmm.18022

**Published:** 2023-11-06

**Authors:** Ya‐Wen Wang, Wen‐Jie Zhu, Ran‐Ran Ma, Ya‐Ru Tian, Xu Chen, Peng Gao

**Affiliations:** ^1^ Department of Pathology Qilu Hospital of Shandong University Jinan Shandong China; ^2^ Key Laboratory for Experimental Teratology of Ministry of Education, Department of Pathology, School of Basic Medical Sciences Shandong University Jinan Shandong China; ^3^ Department of Radiation Oncology, Shandong Cancer Hospital and Institute Shandong First Medical University and Shandong Academy of Medical Science Jinan Shandong China

**Keywords:** CREB1, gastric cancer, lncRNA, PIN1, PIN1P1, pseudogene, YBX1

## Abstract

Long noncoding RNAs (lncRNAs) play critical roles in the carcinogenesis and progression of cancers. However, the role and mechanism of the pseudogene lncRNA PIN1P1 in gastric carcinoma remain unclear. The expression and effects of lncRNA PIN1P1 in gastric cancer were investigated. The transcriptional regulation of CREB1 on PIN1P1 was determined by ChIP and luciferase assays. The mechanistic model of PIN1P1 in gastric cancer was further explored by RNA pull‐down, RIP and western blot analysis. PIN1P1 was overexpressed in gastric cancer tissues, and upregulated PIN1P1 predicted poor prognosis in patients. CREB1 was directly combined with the promoter region of PIN1P1 to promote the transcription of PIN1P1. CREB1‐mediated enhanced proliferation, migration and invasion could be partially reversed by downregulation of PIN1P1. Overexpressed PIN1P1 promoted the proliferation, migration and invasion of gastric cancer cells, whereas decreased PIN1P1 showed the opposite effects. PIN1P1 directly interacted with YBX1 and promoted YBX1 protein expression, leading to upregulation of PIN1, in which E2F1 may be involved. Silencing of YBX1 during PIN1P1 overexpression could partially rescue PIN1 upregulation. PIN1, the parental gene of PIN1P1, was elevated in gastric cancer tissues, and its upregulation was correlated with poor patient outcomes. PIN1 facilitated gastric cancer cell proliferation, migration and invasion. To sum up, CREB1‐activated PIN1P1 could promote gastric cancer progression through YBX1 and upregulating PIN1, suggesting that it is a potential target for gastric cancer.

## 1 INTRODUCTION

Gastric carcinoma is one of the most prevalent and lethal cancers globally.^1^ Despite emerging improvements in gastric cancer therapies, including immunotherapy and targeted therapy, the prognosis of patients remains far from satisfactory.^2^ A comprehensive understanding of the mechanisms of gastric cancer development and progression would be helpful in identifying effective therapeutic targets and improving the outcomes of patients with gastric cancer.

Long noncoding RNAs (lncRNAs) refer to noncoding RNAs with a length > 200 nucleotides, which play crucial roles in the diagnosis and progression of tumours.^3^, ^4^ LncRNAs participate in numerous biological processes, and their abnormal amount and structure play an important role in carcinogenesis, metastasis and chemotherapy resistance.^5^ Pseudogenes, as types of lncRNAs, can function in multiple ways, including binding and titrating molecular regulators that interact with the functional copies.^6^ A PTEN pseudogene expressing the lncRNA PTENpg1 regulates the transcription and translation of PTEN.^7^ LncRNA derived from the pseudogene WFDC21P bound directly to GTPase Ran and enhanced gastric cancer progression through the Akt/GSK3β/β‐catenin pathway.^8^

PIN1 (Peptidylprolyl cis/trans isomerase, NIMA‐interacting 1) is a member of the parvulin subfamily and is known to bind the phosphorylated serine/threonine–proline (pSer/Thr–Pro) motifs of a target protein and then alter its protein structure, stability, subcellular localization and biological functions.^9^ PIN1 is commonly upregulated in malignancies and contributes to the progression and development of tumours, suggesting that PIN1 represents a promising target for cancer.^10^ PIN1P1 (PIN1 pseudogene 1), also known as PIN1L, has been identified as a pseudogene of PIN1.^11^ However, the expression and function of PIN1P1 in human diseases, including cancers, and the effect of PIN1P1 on its parental gene PIN1 have not yet been documented.

Differentially expressed lncRNAs in gastric cancer tissues have been identified by our research team (GEO database GSE72307),^12^, ^13^, ^14^, ^15^, ^16^, ^17^ in which PIN1P1 showed higher expression in gastric cancer tissues than normal mucosa. Here, we investigated a novel lncRNA, PIN1P1, upregulated in gastric cancer and transcriptionally activated by CREB1. PIN1P1 was identified to directly bind to the YBX1 protein, which may mediate PIN1 upregulation and promote gastric cancer progression.

## 2 MATERIALS AND METHODS

### 2.1 Tissue samples

Sixty gastric cancer tissues and eight normal mucosas were collected from patients who underwent surgery at Qilu Hospital of Shandong University and Shandong Provincial Hospital (Jinan, Shandong, China). The fresh tissues were immediately snap‐frozen in liquid nitrogen after being dissected from resected specimens. This study was approved by the Ethic Committee of Shandong University (Jinan, Shandong, China). Written informed consent was obtained from all patients. The work was carried out in accordance with The Code of Ethics of the World Medical Association (Declaration of Helsinki).

### 2.2 Cell lines and cell transfection

Gastric cancer cell lines AGS, MKN45 and BGC823 were obtained from the Chinese Academy of Sciences. Overexpression plasmids of PIN1P1 (pcDNA3.1(+)‐PIN1P1), CREB1 (pcDNA3.1(+)‐CREB1) or control plasmid pcDNA3.1(+) were transfected using TurboFect (Thermo Fisher). Antisense oligonucleotides (ASOs) targeting PIN1P1 (PIN1P1‐ASO; RiboBio) and siRNAs targeting CREB1 or YBX1 (Genepharma) were transfected with INTERFERin (Polyplus transfection).

### 2.3 Real‐time quantitative polymerase chain reaction (RT‐qPCR)

Briefly, total RNA was extracted from gastric cancer tissues or cells by Trizol (Invitrogen). HiScript II Q Select RT SuperMix for qPCR (+gDNA Wiper) (Vazyme, #R233) was used to reverse RNA samples to cDNA. The gDNA wiper Mix in this kit was utilized to remove of genomic DNA. No RT Control Mix in this kit was utilized to confirm the complete removal genomic DNA. ChamQ SYBR qPCR Master Mix (Vazyme, #Q311) was used to detect RNA expression levels by qPCR with a Bio‐Rad CFX 96 Real‐Time System (Bio‐Rad). Primer sequences were provided in Table S1. The specificity of the PIN1P1 and PIN1 primers was verified by the NCBI Primer‐BLAST tool and Sanger‐based sequencing of the qPCR products (Figure S1).

### 2.4 RNA nuclear/cytoplasmic isolation assay

RNA was extracted from the cytoplasm and nucleus separately, according to the instructions of the Nuclear/Cytoplasmic Isolation kit (Biovision). The expression of PIN1P1 in the cytoplasm and nucleus was then detected by RT‐qPCR. GAPDH and U6 were used as controls.

### 2.5 Actinomycin D chase assay

Cells were exposed to DMSO or 2 μg/mL actinomycin D (Sigma‐Aldrich, USA) for 15 min, 30 min, 1 h, 2 h, 3 h and 6 h. Then the expression of c‐Myc mRNA (control) and PIN1P1 was detected using RT‐qPCR.

### 2.6 Proliferation assays

The Cell Counting Kit 8 (CCK8) assay (TargetMol) was used to detect the cells proliferation ability. In brief, cells (5 × 10^3^ cells/well) were inoculated in a 96‐well plate for 24, 48, 72 and 96 h, respectively, and 10 μL of CCK8 solution was added to each well. The plate was incubated for an additional 2 h before measuring the absorbance at the 450 nm wavelength.^18^ For the EdU proliferation assay, the analysis was carried out using the Cell‐Light EdU Apollo 567 In Vitro Kit (Ribobio).^19^ Cells were seeded onto 96‐well plates at 24 h after transfection. After EdU incubation, the cells were treated with an Apollo reaction cocktail, stained with DAPI and visualized under a fluorescent microscope (Olympus). The proliferation rate was determined by calculating the percentage of EdU‐positive cells.

### 2.7 Migration and invasion assays

The migration and invasion assays were performed on gastric cancer cells as previously described.^2^ The migration assay was conducted with Transwell inserts (24‐well format; Corning). For the invasion assay, the previously mentioned inserts were pre‐coated with Matrigel matrix Matrigel (Corning Corp.). The cells (1 × 10^5^) were resuspended in serum‐free medium and seeded in the upper chamber. The lower chambers were filled with complete medium containing 10% FBS. After incubation at 37°C for 24 h, the migrated cells present on the lower side of the membrane were fixed, stained and counted. Crystal violet‐stained cells were counted in five fields from each well at a magnification of 200×.

### 2.8 Chromatin immunoprecipitation (ChIP)

The EZ‐Magna ChIP kit (Millipore) was used for the chromatin immunoprecipitation (ChIP) assay. Cell lysates were immunoprecipitated with anti‐CREB1 antibody (Cell Signaling Technology [CST], #9197, rabbit monoclonal) or IgG antibody. RT‐qPCR primers were designed based on the binding sites of the CREB1 and PIN1P1 promoter regions obtained by ChIP‐seq. The immunoprecipitated promoter regions of PIN1P1 were quantified by RT‐qPCR.

### 2.9 RNA immunoprecipitation (RIP) assay

The RIP assay was performed using the EZ‐Magna RIP RNA‐Binding Protein Immunoprecipitation Kit (Millipore). Briefly, protein A/G magnetic beads were pretreated with YBX1 antibody (ab76149; Abcam) or IgG antibody and then incubated with cell lysate overnight at 4°C. Subsequently, the beads were eluted using a magnetic frame. Finally, co‐precipitated RNAs were extracted, and PIN1P1 enrichment was detected by RT‐qPCR.

### 2.10 RNA pull‐down

RNA pull‐down was performed as previously described.^16^ Briefly, RNA‐bound proteins were eluted and isolated by SDS/PAGE, and silver staining was performed to visualize PIN1P1‐specific bands. Mass spectrometry (Thermo Q Exactive Orbitrap) was used to identify PIN1P1‐associated binding proteins.

### 2.11 Luciferase assays

Luciferase activities were evaluated using the Dual Luciferase Assay Kit (Promega).^20^ The potential CREB1 binding regions of the PIN1P1 promoter were PCR amplified and were then inserted into the NheI/HindIII sites upstream of the firefly luciferase in the pGL3‐Basic vector. The reporter vector pGL3‐Basic, along with the CREB1 expression plasmid or negative control, were transfected into cells.

### 2.12 Western blot

Western blot was conducted as previously described.^21^ The primary antibodies were YBX1 (1:1000, ab76149; Abcam), YBX2 (1:5000, ab154829; Abcam), PHB2 (1:10000, ab182139; Abcam), HER2 (1:1000, #2165; CST), FAK (1:5000, ET1602‐25; HuaAn Biotechnology), E2F1 (1:1000, #3742; CST), PIN1 (1:2000, 10,495‐1‐AP; Proteintech), AKT (1:1000, ab8805; Abcam), c‐Myc (1:1000, #5605; CST), β‐catenin (1:1000, #8480; CST), Cyclin E1 (1:1000, #20808; CST), p27 (1:1000, #3686; CST), β‐actin (1:1000, BA2305; Boster) or GAPDH (1:5000, AB0038; Abways).

### 2.13 Statistical analysis

GraphPad Prism was used for statistical analysis. Data differences between two groups were analysed using a nonparametric test. One‐way anova was used to analyse data differences among the three groups. The web‐based survival analysis tool Kaplan–Meier Plotter^22^, ^23^, ^24^ was used to determine the relationship between PIN1P1 or PIN1 expression and the outcome of patients with gastric cancer. *p*‐values < 0.05 were considered statistically significant.

## 3 RESULTS

### 3.1 PIN1P1 was overexpressed in gastric cancer and correlated with poor prognosis

**FIGURE 1:**
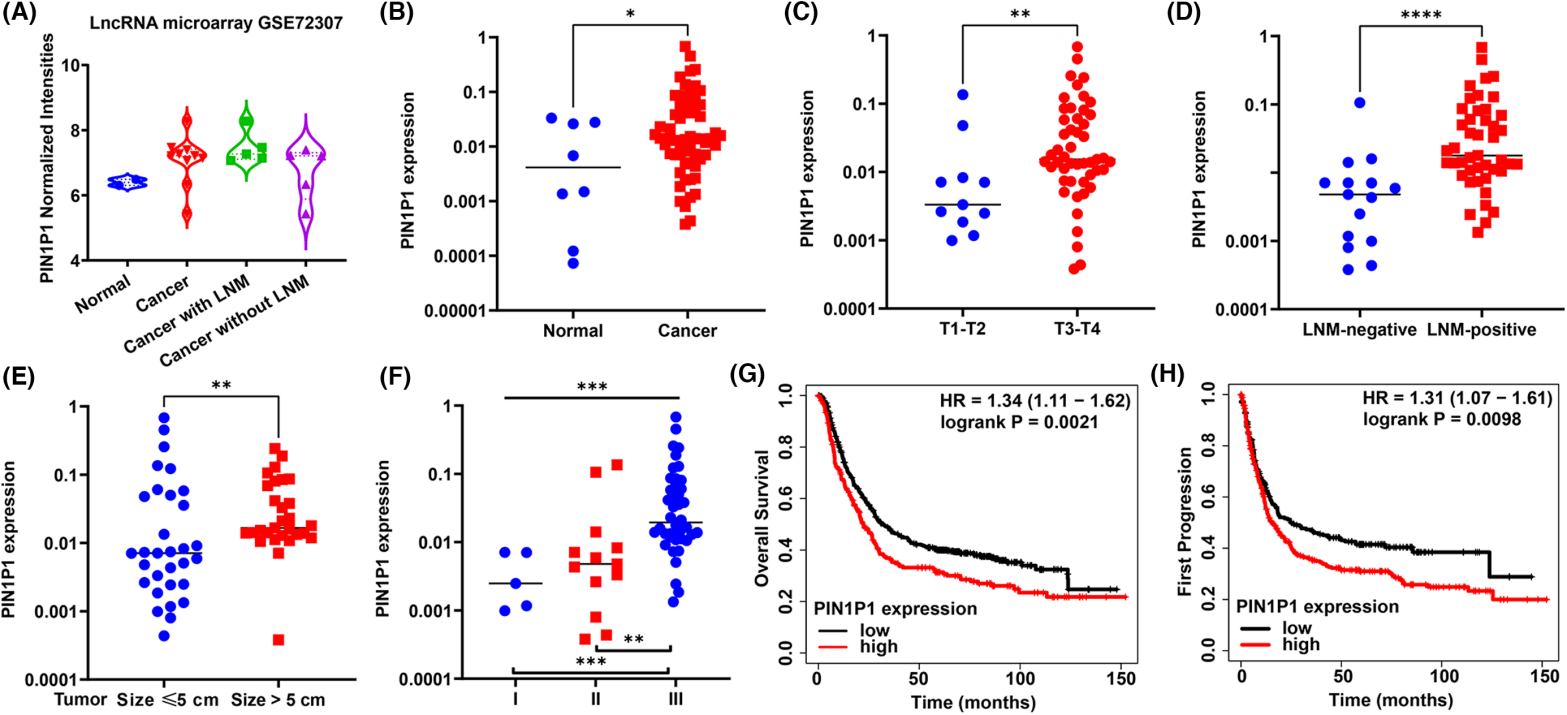
PIN1P1 was upregulated in gastric cancer tissues and predicted a poor outcome. (A) We previously performed a lncRNA microarray to screen gastric cancer‐related lncRNAs (GEO database GSE72307). In the microarray, 10 cases of gastric cancer tissues (five with lymph node metastasis and five without lymph node metastasis) and two cases of normal gastric mucosa tissues were collected in Qilu Hospital of Shandong University. Differential lncRNA screening was performed using the Human Gene Expression Microarray (Human 8 × 60 K LncRNA Microarray v2.0; Arraystar). PIN1P1 was overexpressed in gastric cancer tissues (*n* = 10) compared to normal gastric mucosa (*n* = 2). PIN1P1 expression tended to be even higher in cancer tissues with lymph node metastasis (LNM) (*n* = 5) than in those without (*n* = 5). (B) PIN1P1 was increased in gastric cancer tissues (*n* = 60) compared to normal gastric mucosa (*n* = 8, Mann–Whitney test), as determined by RT‐qPCR. The horizontal line represents the median of each group. (C–E) PIN1P1 was upregulated in gastric cancer tissues with deeper invasion depth (C), positive LNM (D) and larger tumour size (E) (Mann–Whitney test). (F) PIN1P1 showed a stepwise pattern of upregulation from tumour stage I to stages II and III (One‐way anova). (G, H) The Kaplan–Meier plotter database (http://kmplot.com/analysis/index.php?p=service&cancer=gastric) demonstrated that patients with high expression of PIN1P1 showed poorer overall survival (G, HR = 1.34, *p* = 0.0021) and rapid progression (H, HR = 1.31, *p* = 0.0098) than those with low PIN1P1 expression. (*p < 0.05, **p < 0.01, ***p < 0.001).

We previously conducted a lncRNA microarray to screen lncRNAs that were differentially expressed in gastric cancer (GEO GSE72307).^12^, ^13^, ^14^, ^15^, ^16^, ^17^ Compared to the normal gastric mucosas, PIN1P1 was increased in gastric cancer tissues, especially in those with lymph node metastasis (LNM) (Figure 1A). PIN1P1 is the pseudogene of the critical oncogene PIN1, but has not yet been investigated in human cancer. Here, the expression of PIN1P1 was analysed in another 60 cases of gastric cancer tissues and eight normal gastric mucosas through RT‐qPCR. PIN1P1 was upregulated in gastric cancer tissues compared to normal gastric mucosas (Figure 1B). Higher expression of PIN1P1 was found in cancer tissues with deeper invasion depth (Figure 1C, T3–T4 vs. T1–T2; Table 1), positive LNM status (Figure 1D; Table 1), larger tumour size (Figure 1E; Table 1) and advanced tumour stage (Figure 1F; Table 1). High expression of PIN1P1 was associated with worse overall survival (Figure 1G) and rapid progression (Figure 1H) in patients with gastric cancer.

**TABLE 1:** Association between PIN1P1 or PIN1 expression and clinicopathological parameters in gastric cancer.

Variables	n	PIN1P1 expression^a^		*p* Value	PIN1 expression^a^		*p* Value
		Low	High		Low	High	
Age (y)							
≤60	25	11	14		13	12	
>60	34	19	15	0.4348	17	17	1.0000
Missing	1						
Sex							
Female	12	7	5		6	6	
Male	48	23	25	0.7480	24	24	1.0000
Tumour size							
≤5 cm	31	21	10		19	12	
>5 cm	29	9	20	0.0092	11	18	0.1205
Depth of invasion (T stage)							
T1 + T2	11	9	2		6	5	
T3 + T4	49	21	28	0.0419	24	25	1.0000
Lymph node metastasis							
Negative	15	12	3		13	2	
Positive	45	18	27	0.0153	17	28	0.0021
N stage							
N0	15	12	3		13	2	
N1	7	2	5		3	4	
N2	15	8	7		8	7	
N3	23	8	15	0.0309	6	17	0.0036
Tumour stage							
I	5	5	0		5	0	
II	13	10	3		8	5	
III	42	15	27	0.0022	17	25	0.0271

^a^ According to the median value of PIN1P1 or PIN1 expression, patients were divided into high and low expression groups.

As PIN1P1 lies in the intron of the protein‐coding gene LRRC7, we also checked the expression of these two transcripts in gastric cancer samples through the GEPIA database (http://gepia.cancer‐pku.cn/detail.php). It was shown that PIN1P1 was weakly positively correlated with LRRC7 expression (Figure S2).

Actinomycin D chase assay (with short half‐life c‐Myc mRNA as a control) is usually used to show the stability of lncRNA.^4^, ^13^, ^16^, ^25^ The results of the actinomycin D chase assay showed that PIN1P1 was more stably expressed than short half‐life c‐Myc mRNA in gastric cancer cells (Figure S3A,B), suggesting the possibility of PIN1P1 as a stable biomarker in gastric cancer. The subcellular distribution of PIN1P1 was determined, and PIN1P1 was mainly found in the nucleus of gastric cancer cells (Figure S3C–E).

### 3.2 CREB1 activated PIN1P1 transcription in gastric cancer cells

**FIGURE 2:**
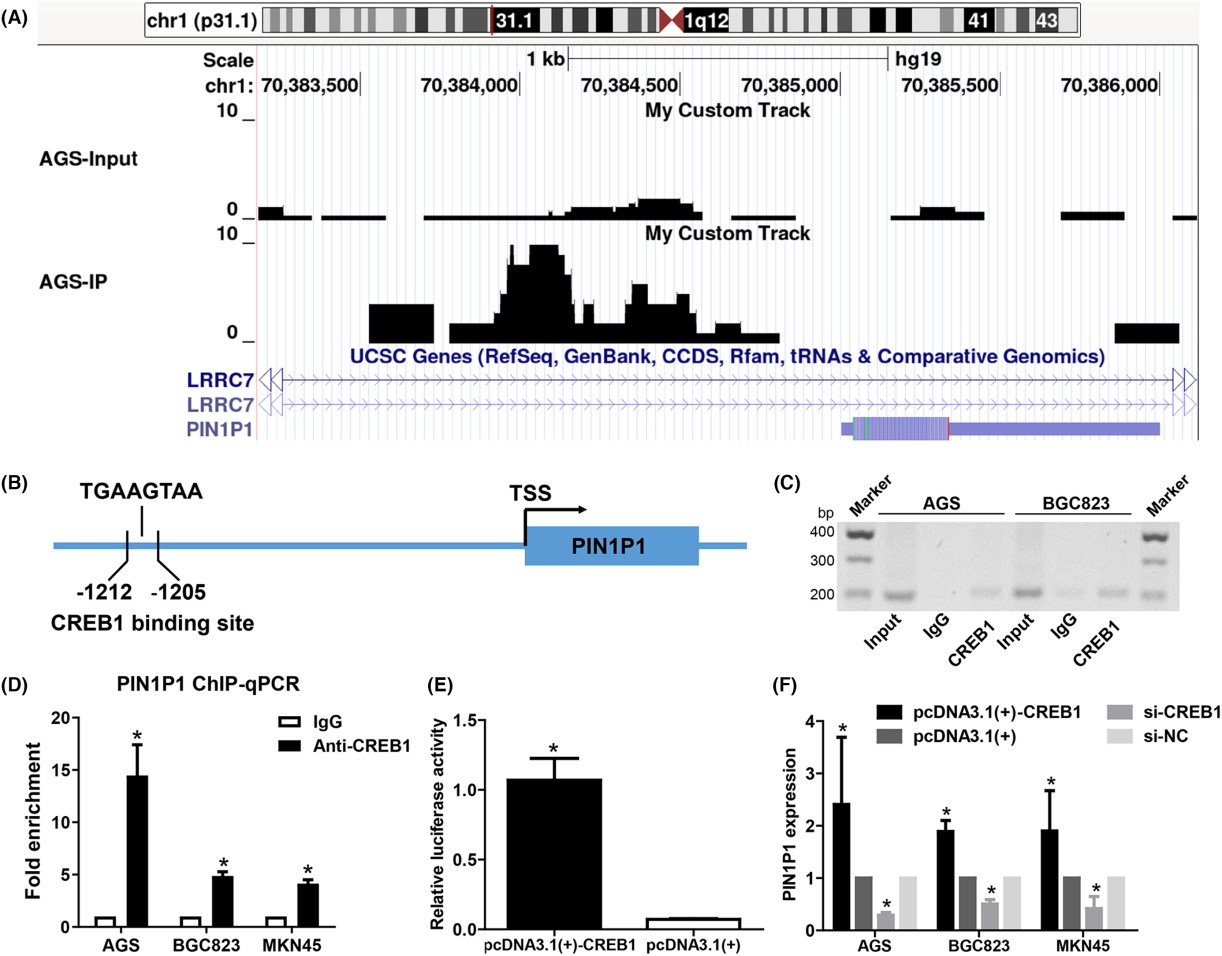
CREB1 is bound to the promoter region of PIN1P1 to promote PIN1P1 expression. (A) Genome Browser tracks of the Chip‐Seq tags surrounding the PIN1P1 promoter. ChIP‐seq analysis (GEO database GSE220708) in AGS gastric cancer cells indicated that CREB1 was enriched in the promoter region of PIN1P1. (B) A schematic representation of the PIN1P1 locus and the CREB1 binding sites predicted by JASPAR software in the promoter region. (C, D) ChIP‐PCR (C) and ChIP‐qPCR (D) showed that CREB1 was enriched in the promoter of PIN1P1. For the sake of a better visualization of the band, an inverted image was shown (C). (E) The luciferase assay revealed that CREB1 could reinforce luciferase activity, suggesting that CREB1 could activate the PIN1P1 promoter region. (F) Expression of PIN1P1 was upregulated after overexpression of CREB1, while the expression of PIN1P1 was repressed with CREB1 downregulation (*p < 0.05). Each treatment was three times replicated, and the Mann–Whitney test was used for comparison between the two groups.

We previously performed a ChIP‐seq analysis to elucidate transcriptional factor CREB1 binding in AGS gastric cancer cells (GEO database GSE220708). Genome Browser tracks of the Chip‐Seq tags displayed that CREB1 could combine with the putative promoter of PIN1P1 (Figure 2A), suggesting that CREB1 may be involved in the regulation of PIN1P1. The JASPAR database showed the potential binding sites of CREB1 in the PIN1P1 promoter (a schematic representation, Figure 2B). ChIP‐PCR (Figure 2C) and ChIP‐qPCR (Figure 2D) assays showed that CREB1 is directly bound to the promoter of PIN1P1. CREB1 clearly increased the promoter activities of PIN1P1, as revealed by the results of the luciferase assay (Figure 2E). The RT‐qPCR assay confirmed that CREB1 could upregulate the expression of PIN1P1, while CREB1 knockdown downregulated the expression of PIN1P1 (Figure 2F; Figure S4). Furthermore, the expression of CREB1 in gastric cancer tissues was positively related to PIN1P1 expression (Figure S5A, Spearman *r* = 0.5168, *p* < 0.0001).

### 3.3 PIN1P1 facilitated gastric cancer cell proliferation, migration and invasion

**FIGURE 3:**
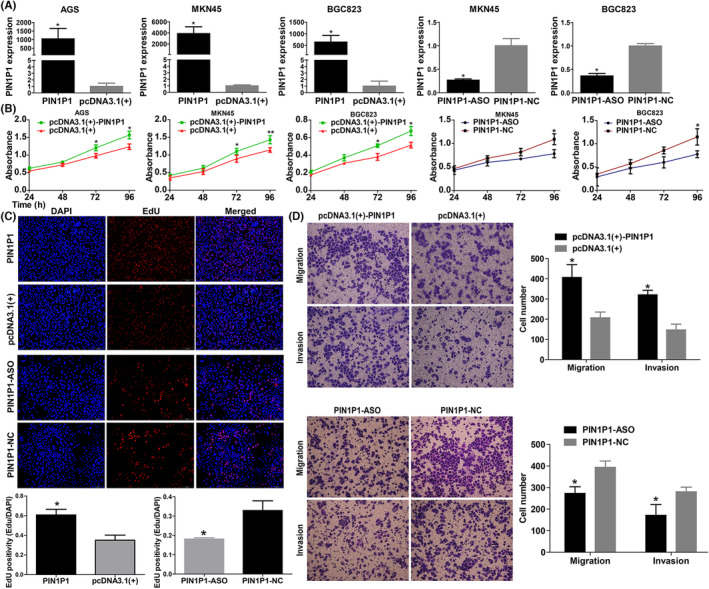
PIN1P1 promoted gastric cancer proliferation, migration and invasion. (A) The overexpression plasmid (PIN1P1) significantly upregulated the expression of PIN1P1. Transfection of PIN1P1 with ASO successfully reduced the expression of PIN1P1. (B) CCK8 assays demonstrated that PIN1P1 overexpression accelerated cell proliferation, while knockdown of PIN1P1 inhibited cell proliferation in BGC823 cells. (C) Results of EdU assays indicated that upregulation or downregulation of PIN1P1 in BGC823 cells could enhance or repress cell proliferation, respectively. (D) PIN1P1 overexpression enhanced in BGC823 cells migration and invasion, whereas silencing of PIN1P1 suppressed cell migration and invasion (magnification, ×200; *p < 0.05). Each experiment was replicated three times, and Mann–Whitney test was used for comparison between the two groups.

PIN1P1 was overexpressed by transfection with the PIN1P1 overexpression plasmid or knocked down by the PIN1P1 antisense oligonucleotide (ASO; Figure 3A), with the aim of examining the effect of PIN1P1 on gastric cancer cells. The results of CCK8 and EdU proliferation assays demonstrated that PIN1P1 overexpression enhanced gastric cancer proliferation capabilities, whereas knockdown of PIN1P1 restrained cell proliferation (Figure 3B,C; Figure S6A,B). Additionally, PIN1P1 overexpression markedly enhanced cell migration and invasion, while silencing of PINP1 abolished this effect (Figure 3D; Figure S6C,D).

To clarify the role of PIN1P1 in CREB1‐mediated enhanced proliferation, migration and invasion, we performed rescue experiments and found that inhibiting the expression of PIN1P1 could partially reverse the proliferation, migration and invasion activities induced by CREB1 overexpression in gastric cancer cells (Figure S7).

### 3.4 PIN1P1 interacted with YBX1 and upregulated PIN1

**FIGURE 4:**
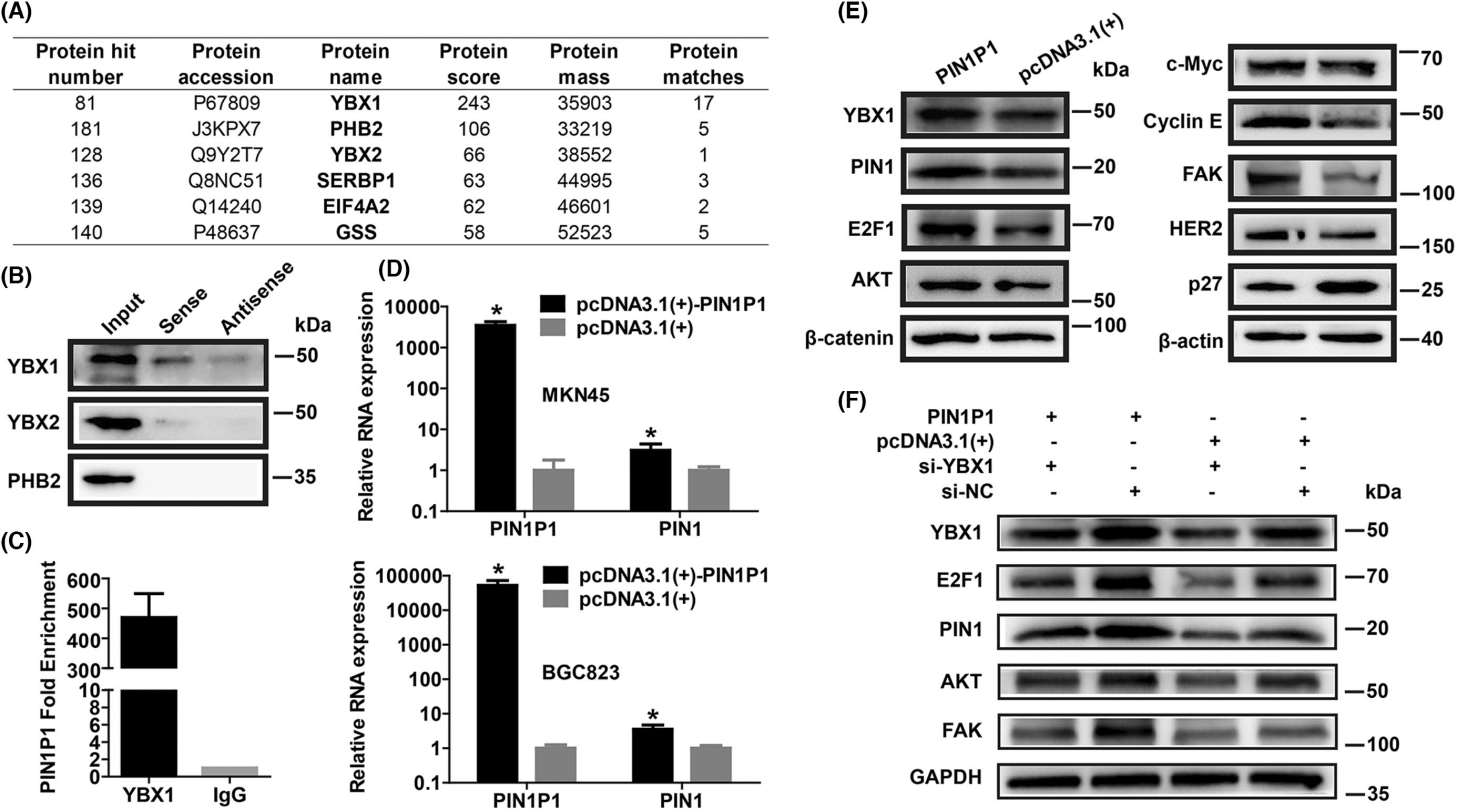
PIN1P1 interacted with YBX1 and upregulated PIN1. (A) Numerous proteins were found to potentially bind with PIN1P1 via RNA pull‐down assay and mass spectrometry. (B) After PIN1P1 pull‐down in BGC823 cells, the binding protein of PIN1P1 was identified by western blot. YBX1, but not YBX2 and PHB2, was identified as a main PIN1P1‐interacting protein. (C) Interaction between YBX1 and PIN1P1 was detected via RIP assay using antibodies against YBX1 or IgG in BGC823 cells. (D) Overexpression of PIN1P1 upregulated PIN1 mRNA expression in gastric cancer cells. (E) Protein expression of YBX1, E2F1, PIN1 and downstream signalling molecules of PIN1 were detected by western blot in BGC823 cells with PIN1P1 overexpression. (F) YBX1 siRNA transfection could inhibit the PIN1P1‐induced protein expression of YBX1, E2F1, PIN1 and PIN1 targets.

RNA pull‐down was conducted to screen proteins interacting with PIN1P1. Mass spectrometry analysis indicated that PIN1P1 binds to many proteins (Figure 4A). Three potential binding proteins, including YBX1, YBX2 and PHB2, were selected for further validation considering their molecular weights, protein scores, subcellular localizations and potential involvement in tumour metastasis and progression. However, only YBX1 was significantly detectable by western blot after the RNA pull‐down of PIN1P1, and thus we picked up YBX1 protein as the main interacting partner of PIN1P1 (Figure 4B). The RIP assay confirmed the specific interaction between PIN1P1 and YBX1 (Figure 4C). Moreover, western blot showed that PIN1P1 could upregulate the expression of YBX1 protein (Figure 4E).

We are interested in the effect of PIN1P1 on the parental PIN1, and it was found that PIN1P1 overexpression could significantly upregulate PIN1 (Figure 4D). PIN1P1 expression in gastric cancer tissues showed a positive relationship with PIN1 expression (Figure S5B, Spearman *r* = 0.5079, *p* < 0.0001). YBX1 has been shown to transactivate E2F1,^26^, ^27^ and interestingly, E2F1 could transcriptionally induce PIN1 expression.^28^, ^29^, ^30^ Western blot confirmed that PIN1P1 transfection upregulated E2F1 and PIN1 protein (Figure 4E). As expected, the downstream signalling targets of PIN1, including AKT, β‐catenin, Cyclin E, c‐Myc, FAK and HER2, were upregulated, and p27 was downregulated by PIN1P1 (Figure 4E). To test the dependence of YBX1 on PIN1P1‐induced PIN1 upregulation, co‐transfection of YBX1 siRNA during PIN1P1 overexpression was performed. It was found that YBX1 siRNA treatment could partially reverse the PIN1P1‐mediated upregulation of YBX1, E2F1, PIN1 and the PIN1 targets during PIN1P1 overexpression (Figure 4F).

### 3.5 PIN1 was overexpressed in gastric cancer and promoted cell proliferation, migration and invasion

**FIGURE 5:**
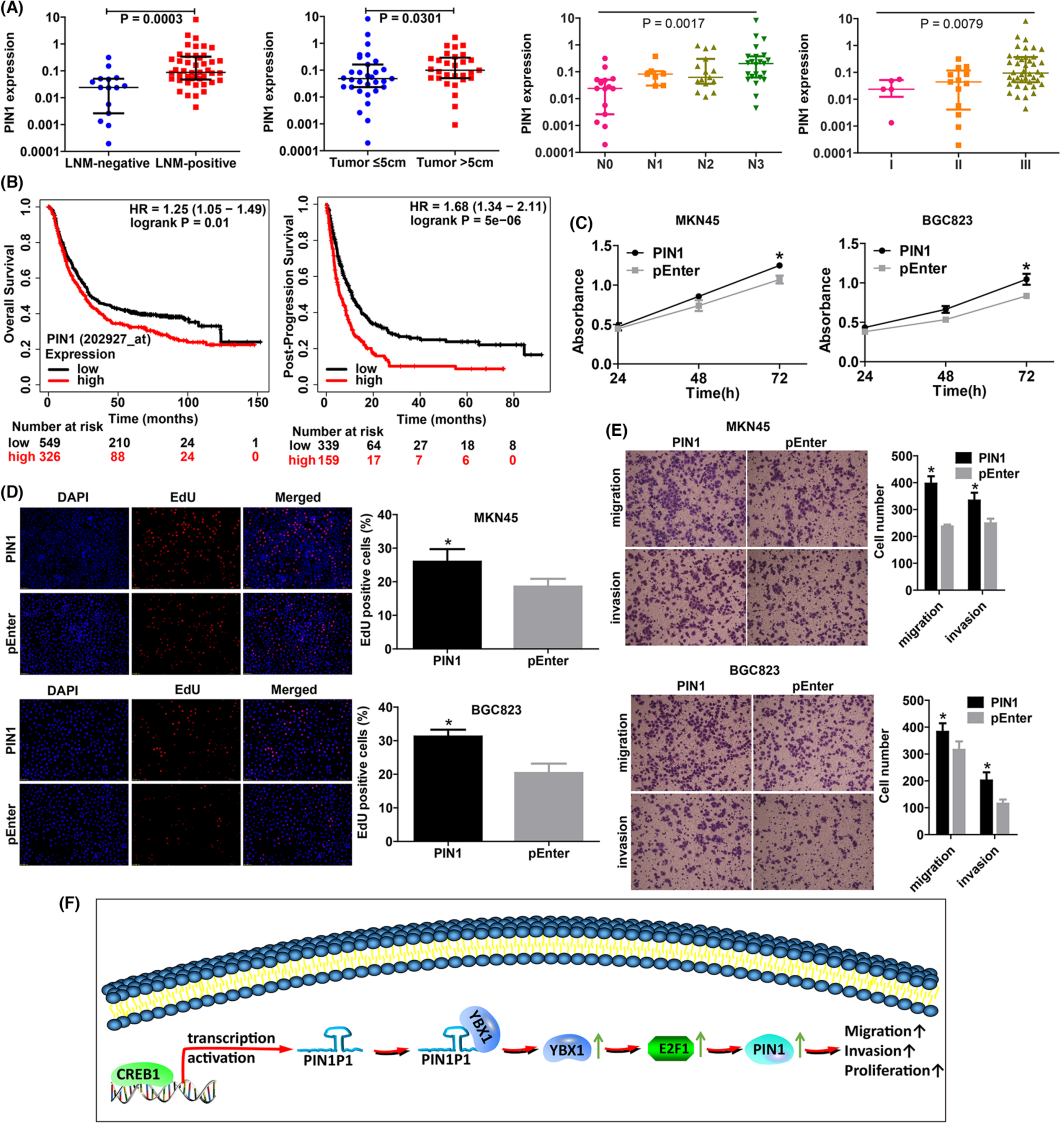
PIN1 was associated with aggressive clinicopathological parameters and promoted cell proliferation, migration and invasion. (A) PIN1 was upregulated in gastric cancer tissues with LNM, larger tumour size and advanced N stage and tumour stage. (B) The Kaplan–Meier plotter database (http://kmplot.com/analysis/index.php?p=service&cancer=gastric) revealed that high expression of PIN1 was correlated with poorer overall survival and post‐progression survival in patients with gastric cancer. (C) CCK8 assays indicated that PIN1 upregulation remarkably accelerated cell proliferation. (D) The EdU assay revealed that PIN1 overexpression significantly enhanced cell proliferation. (E) Overexpression of PIN1 promoted cell migration and invasion (magnification, ×200). Each treatment was three times replicated, and Mann–Whitney test was used for comparison between the two groups (*p < 0.05). (F) A mechanistic model of PIN1P1 in gastric cancer is shown. CREB1 activates PIN1P1 transcription by directly binding to its promoter. PIN1P1 interacted with YBX1 and promoted YBX1 protein expression, leading to upregulation of PIN1, which may involve E2F1.

As we have shown that PIN1P1 could promote PIN1 expression, we next explored the expression of PIN1 in gastric cancer tissues and its function in gastric cancer cells. PIN1 was significantly elevated in tissues with LNM compared to those without (Figure 5A; Table 1). Higher expression of PIN1 was found in cancer tissues with larger tumour sizes (Figure 5A). PIN1 was stepwise upregulated with N stage and tumour stage (Figure 5A; Table 1). High expression of PIN1 was correlated with poorer overall survival and post‐progression survival in patients with gastric cancer (Figure 5B).

To evaluate the biological effects of PIN1, gain‐of‐function studies were performed by transfection of the PIN1 overexpression vector into gastric cancer cells. EdU and CCK8 proliferation assays indicated that PIN1 enhanced cell proliferation of gastric cancer (Figure 5C,D) and significantly increased gastric cancer cell migration and invasion (Figure 5E).

## 4 DISCUSSION

Increasing evidence has proven the important role of lncRNAs in various tumours, including gastric cancer.^31^ LncRNA Linc01133 promotes gastric cancer growth by increasing YAP1 nuclear location.^12^ LncRNA lnc‐LEMGC binds to DNA‐PKcs and inhibits metastasis of gastric cancer.^16^ However, the potential biological functions and mechanisms of most pseudogene‐derived lncRNAs in gastric cancer remain unknown. Herein, a previously undescribed oncogenic lncRNA derived from the pseudogene PIN1P1 was confirmed to be upregulated in gastric cancer. High expression of PIN1P1 was positively related to LNM, tumour size, tumour stage and the poor outcome of patients with gastric cancer. Moreover, PIN1P1 facilitated proliferation, migration and invasion of gastric cancer cells. LncRNA PIN1P1 is directly combined with the YBX1 protein, which elevated the expression of PIN1 and modulated the downstream targets of PIN1. Furthermore, PIN1P1 was directly activated by the transcription factor CREB1.

At present, few studies have focused on the upstream regulation mechanism of lncRNA. Recent studies have highlighted the role of transcriptional regulation of lncRNAs in gastric cancer. EGR1‐induced lncRNA HNF1A‐AS1 facilitates the gastric cancer cell cycle by sponging miR‐661 and upregulating CDC34.^32^ SP1‐activated lncRNA GCMA sponges miR‐124/34a and stimulates tumour metastasis of gastric cancer.^17^ Our results demonstrated that PIN1P1 could be transcriptionally activated by CREB1 via binding to its promoter region. CREB1 (cAMP response element binding protein 1) is a member of the basic leucine zipper (bZIP) transcription factors and acts as an oncogene in tumour initiation and progression in various tumours.^33^ We have shown CREB1 is overexpressed in gastric cancer and predicts metastasis, advanced stage and poor prognosis.^34^ Previously identified transcriptional targets of CREB1 were mostly protein‐coding genes^33^; however, the noncoding targets of CREB1 remain to be determined. Recently, CREB1 was found to contribute to colorectal cancer cell plasticity through increasing transcription of the oncogenic lncRNA CCAT1 and upregulation of the NF‐κB pathways.^35^ We have previously performed a genome‐wide screening of CREB1 targets by ChIP‐seq in gastric cancer cells (GEO database GSE220708) and found numerous targets of CREB1, in which PIN1P1 was involved. Here, we show that lncRNA PIN1P1 is one of the direct target genes of CREB1 in gastric cancer and is transcriptionally activated by CREB1. And through rescue experiments, we found that PIN1P1 is a functional target of CREB1 in promoting the tumorigenesis of gastric cancer.

The biological function of PIN1P1 in gastric cancer has not been reported previously. It was confirmed that PIN1P1 could promote gastric cancer cell proliferation, migration and invasion, which is consistent with the oncogenic effects of PIN1 in gastric cancer.^36^, ^37^ PIN1 has been proven to promote gastric cancer proliferation and metastasis by activating PI3K/AKT and Wnt/β‐catenin signalling.^37^ Mechanistically, we found that PIN1P1 interacted with YBX1, upregulated its protein expression and heightened PIN1 expression, thereby activating the downstream signalling of PIN1. Most of the PIN1 targets were upregulated during PIN1P1 overexpression; however, p27 was downregulated. p27 is a cyclin‐dependent kinase inhibitor and has been associated with cell cycle and proliferation inhibition.^38^ Reduced expression of p27 is commonly associated with poor prognosis in many malignancies, including gastric cancer.^39^ PIN1 has been revealed to modulate p27 expression, cell cycle and proliferation.^40^ Downregulation of p27 upon PIN1P1 overexpression is consistent with the findings that PIN1P1 could promote cell proliferation in gastric cancer.

To elucidate the mechanism underlying PIN1P1‐induced upregulation of PIN1, we preliminary found that PIN1P1‐mediated YBX1 upregulation could enhance expression of E2F1, a transcriptional activator of PIN1.^28^, ^29^, ^30^ This is consistent with our findings that PIN1P1 could upregulate PIN1 expression at both the mRNA and protein levels. YBX1, also known as DNA‐binding protein (dbpB) and nuclease‐sensitive protein 1 (NSEP1), is a member of the cold shock family of proteins.^41^ YBX1 is an important contributor to tumorigenesis and is correlated with tumour stage and patient prognosis in cancer.^41^ Here we show that PIN1P1 could bind and upregulate the YBX1 protein; however, in‐depth mechanisms are needed in the future. Interestingly, YBX1 expression is associated with the activity of the E2F1 transcription factor,^27^ and PIN1 is controlled by E2F1,^42^ so the potential ‘YBX1‐E2F1‐PIN1’ axis was proposed (Figure 5F). We found that PIN1P1‐induced upregulation of PIN1 is dependent on YBX1. More studies are needed to confirm the current findings.

As expected, we confirmed that PIN1 is upregulated in gastric cancer and promotes cancer progression. PIN1 is considered a potential target for tumours,^43^ and several PIN1 inhibitors and chemotherapeutics have been designed and developed to reduce cell proliferation, repress tumorigenesis or overcome resistance to cancer therapies.^44^, ^45^ PIN1 is upregulated in gastric cancers and is a valuable target in these tumours.^46^ The newly discovered PIN1P1/PIN1 axis may provide new therapeutic approaches for a PIN1‐targeted anticancer strategy. Previous studies have demonstrated that pseudogenes could regulate parental gene expression by various mechanisms, such as miRNA sponging and chromatin remodelling.^47^ For example, PTENP1 was found to upregulate PTEN transcript expression and inhibit cell proliferation and migration through decoying miR‐19b in breast cancer.^48^ Johnsson et al. showed that PTEN pseudogene‐expressed noncoding RNA, PTENpg1, could epigenetically modulate PTEN transcription through the recruitment of DNMT3a and EZH2.^7^ Here we found that in gastric cancer, PIN1P1 interacted with YBX1, upregulating its protein expression, which in turn enhanced the expression of PIN1, in which transcription factor E2F1 may be involved. However, our study is limited to PIN1P1 function in vitro, and the mechanism underlying PIN1P1‐mediated YBX1 upregulation is unclear, which provides directions for future research.

We have also noted that PIN1P1 or PIN1 dysregulation was identified in a retrospective single institution cohort with a relatively small sample size (especially for non‐tumorous samples). Further work is needed, using larger multi‐centre cohorts, ideally with varied geographic and ethnic populations, with different stages, subtypes and treatment regimens, as well as adequate follow‐up, to further elucidate the diagnostic and prognostic value of PIN1P1 and PIN1.

In conclusion, lncRNA PIN1P1 has been identified as an effector of gastric cancer progression and is transcriptionally induced by CREB1. We reveal that the pseudogene PIN1P1 promotes the progression of gastric cancer by interacting with YBX1 and activating PIN1. Our findings offer insights into the mechanisms underlying pseudogene lncRNA‐associated gastric carcinoma progression and lay the foundation for further PIN1P1/PIN1‐targeting therapeutical approaches for gastric cancer.

## AUTHOR CONTRIBUTIONS

**Ya‐Wen Wang:** Data curation (equal); funding acquisition (equal); investigation (equal); resources (equal); writing – original draft (equal). **Wen‐Jie Zhu:** Investigation (equal); resources (equal). **Ran‐Ran Ma:** Investigation (equal); resources (equal). **Ya‐Ru Tian:** Investigation (equal); resources (equal). **Xu Chen:** Conceptualization (equal); funding acquisition (equal); investigation (equal); methodology (equal); resources (equal); supervision (equal); writing – review and editing (equal). **Peng Gao:** Conceptualization (equal); supervision (equal); writing – review and editing (equal).

## FUNDING INFORMATION

This study was supported by the Natural Science Foundation of Shandong Province (No. ZR2020LZL009, ZR2019BH061 and ZR2019BH034) and the National Natural Science Foundation of China (Grant No. 81802406 and 81902698). The sponsors had no roles in study design; in the collection, analysis and interpretation of data; in the writing of the report; and in the decision to submit the article for publication.

## CONFLICT OF INTEREST STATEMENT

None.

## Supporting information


Figures S1‐S7

Table S1

## Data Availability

Microarray data and ChIP‐seq data were deposited in NCBI Gene Expression Omnibus (GEO) database. The original contributions presented in the study are included in the article/Supporting Information. Further inquiries can be directed to the corresponding authors.
