# Supplementary material for: PIN1P1 is activated by CREB1 and promotes gastric cancer progression via interacting with YBX1 and upregulating PIN1
Source: J Cell Mol Med. 2023 Nov 6;28(1):e18022. doi: 10.1111/jcmm.18022 (PMC10805483; doi:10.1111/jcmm.18022)
Supplement: Supplementary file 1 — Figures S1‐S7 [file JCMM-28-e18022-s001.docx]

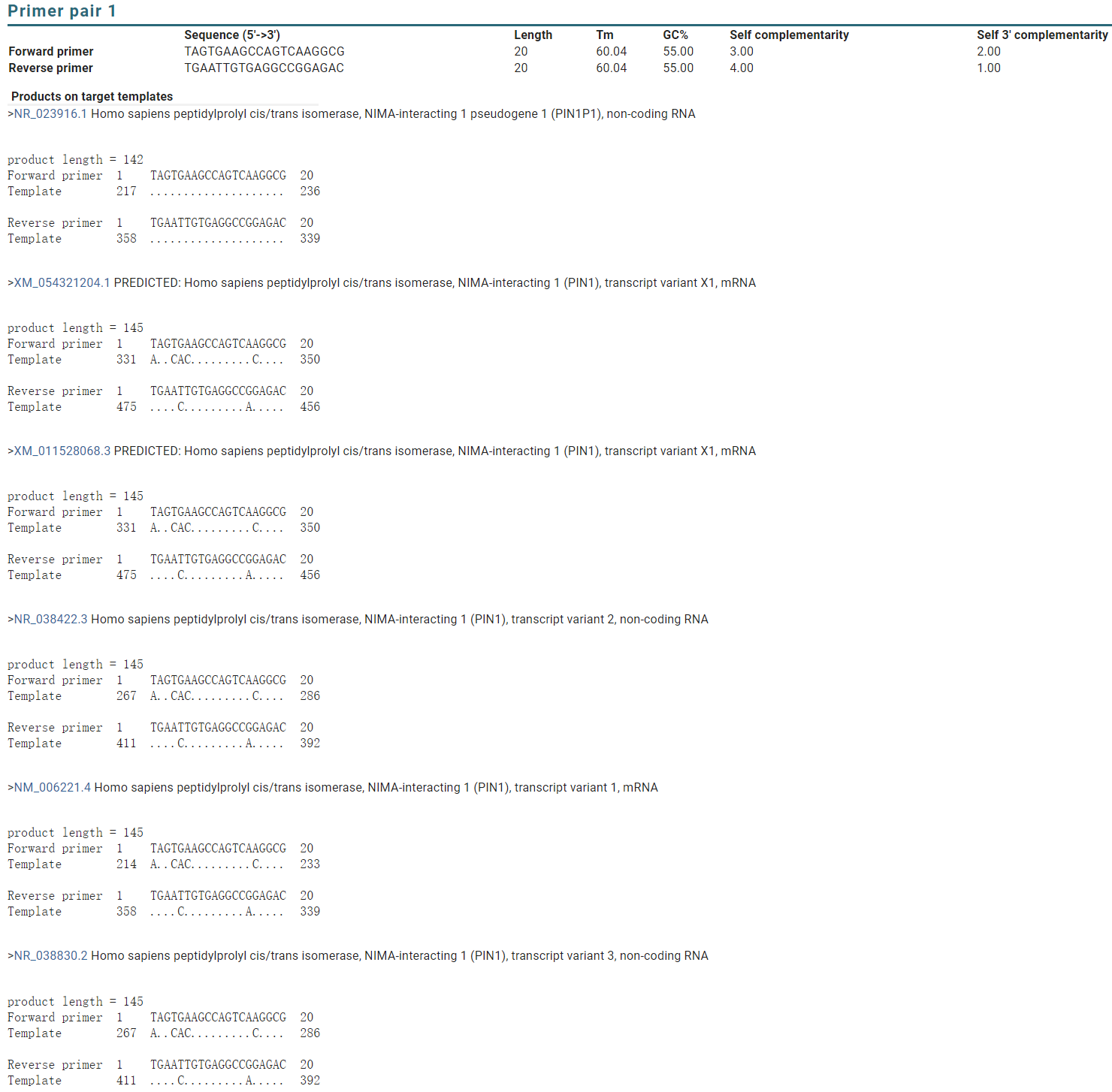


Supplemental Figure S1A


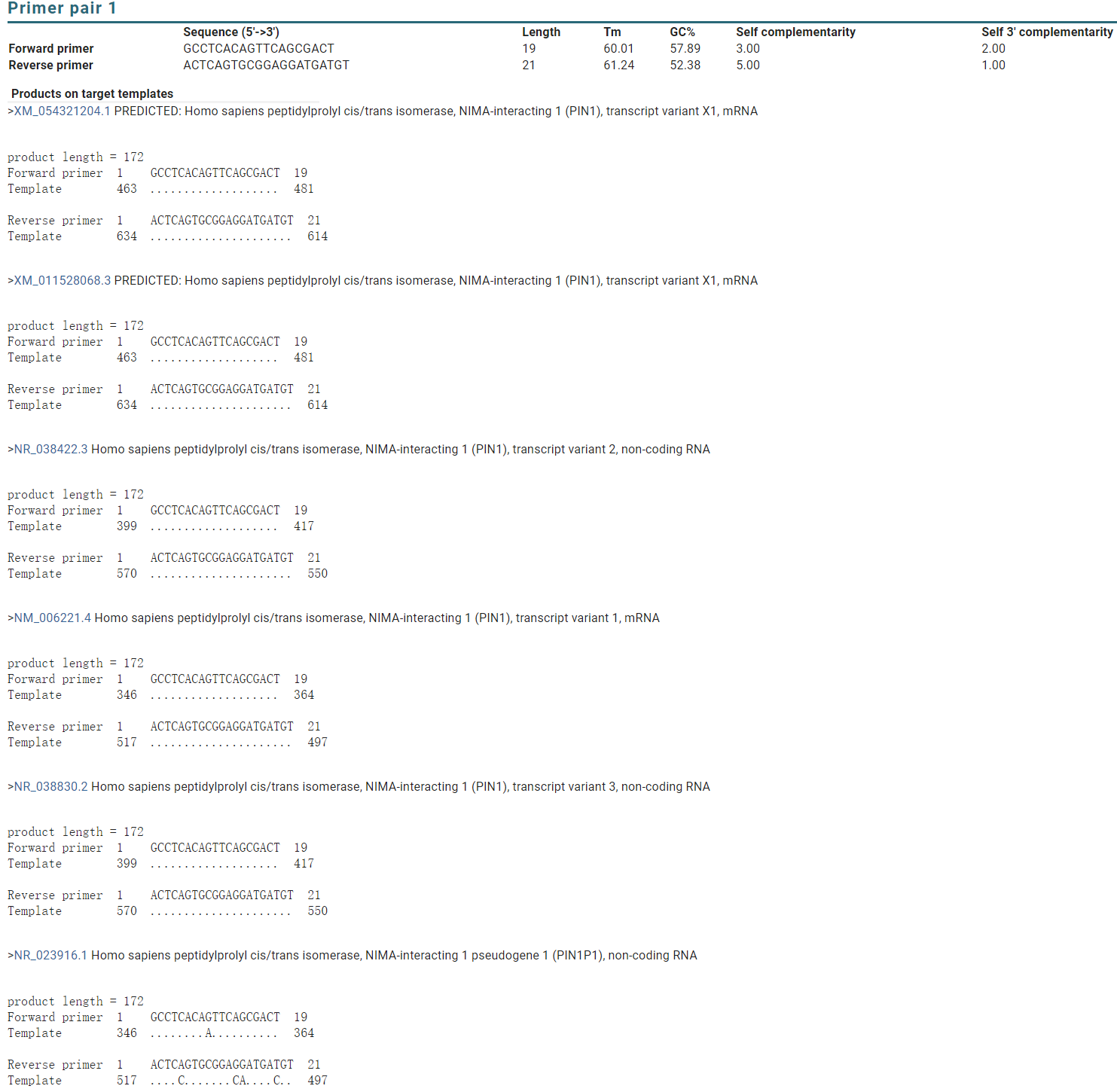


Supplemental Figure S1B


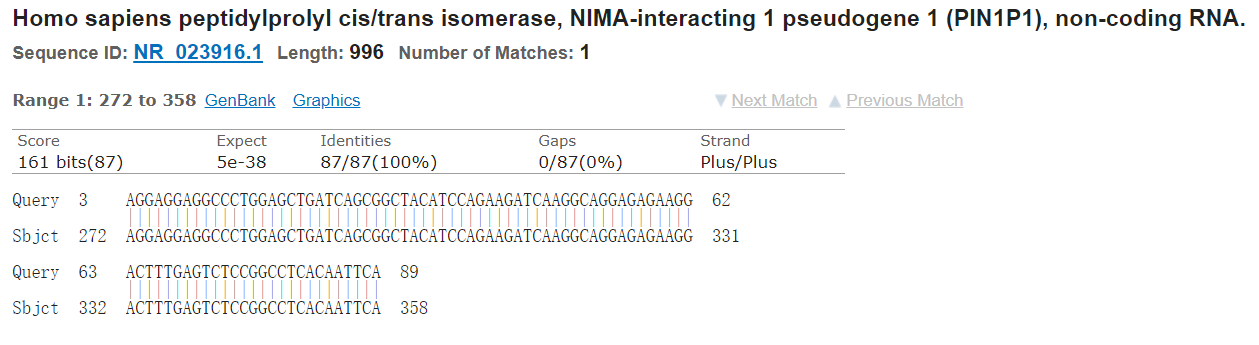


Supplemental Figure S1C


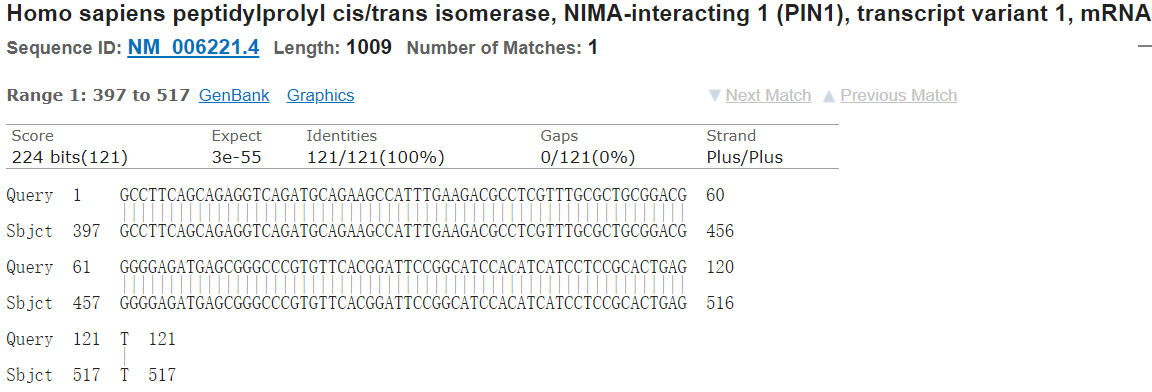


Supplemental Figure S1D


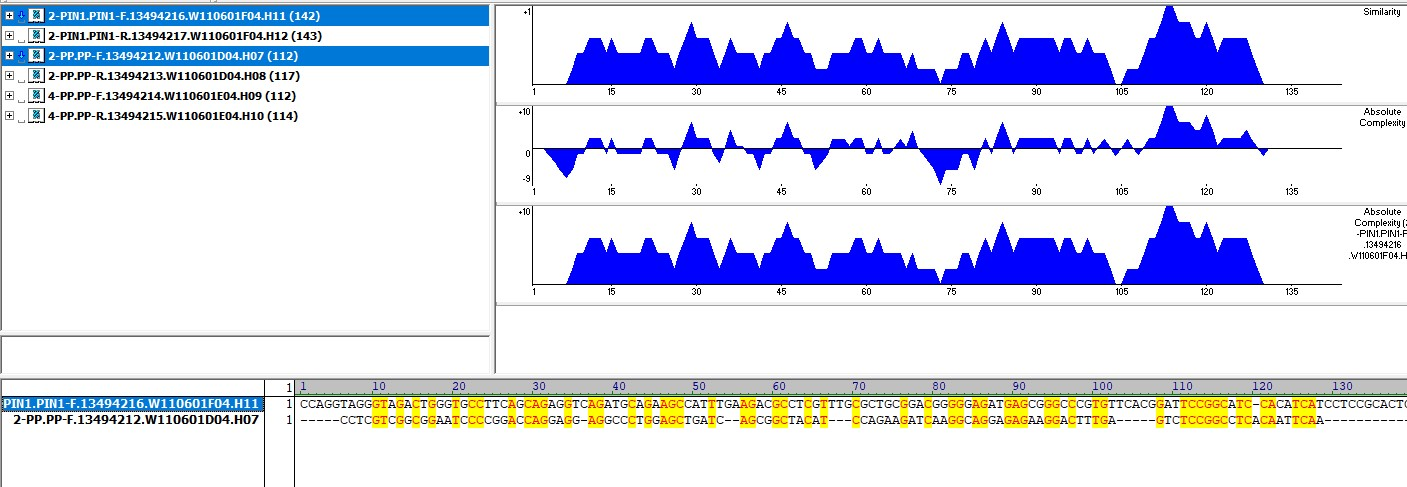


Supplemental Figure S1E


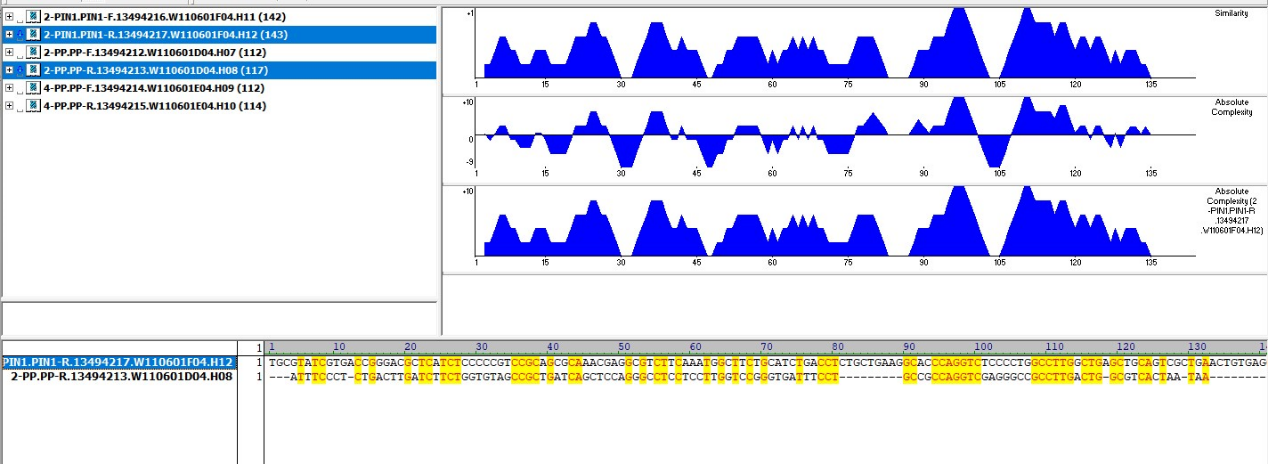


Supplemental Figure S1F

**Supplemental Figure S1. Evidences of primers specificity.** The NCBI Primer-BLAST tool showed that the PIN1P1 and PIN1 primers were completely specific to PIN1P1 and PIN1, respectively (A-B). Sanger-based sequencing of the qPCR products of PIN1P1 and PIN1 primers showed that they were only matched (100%) to PIN1P1 and PIN1, respectively (C-D). Sequence alignment analysis revealed that the two qPCR products (PIN1P1 or PIN1) had different sequences (E-F).

**
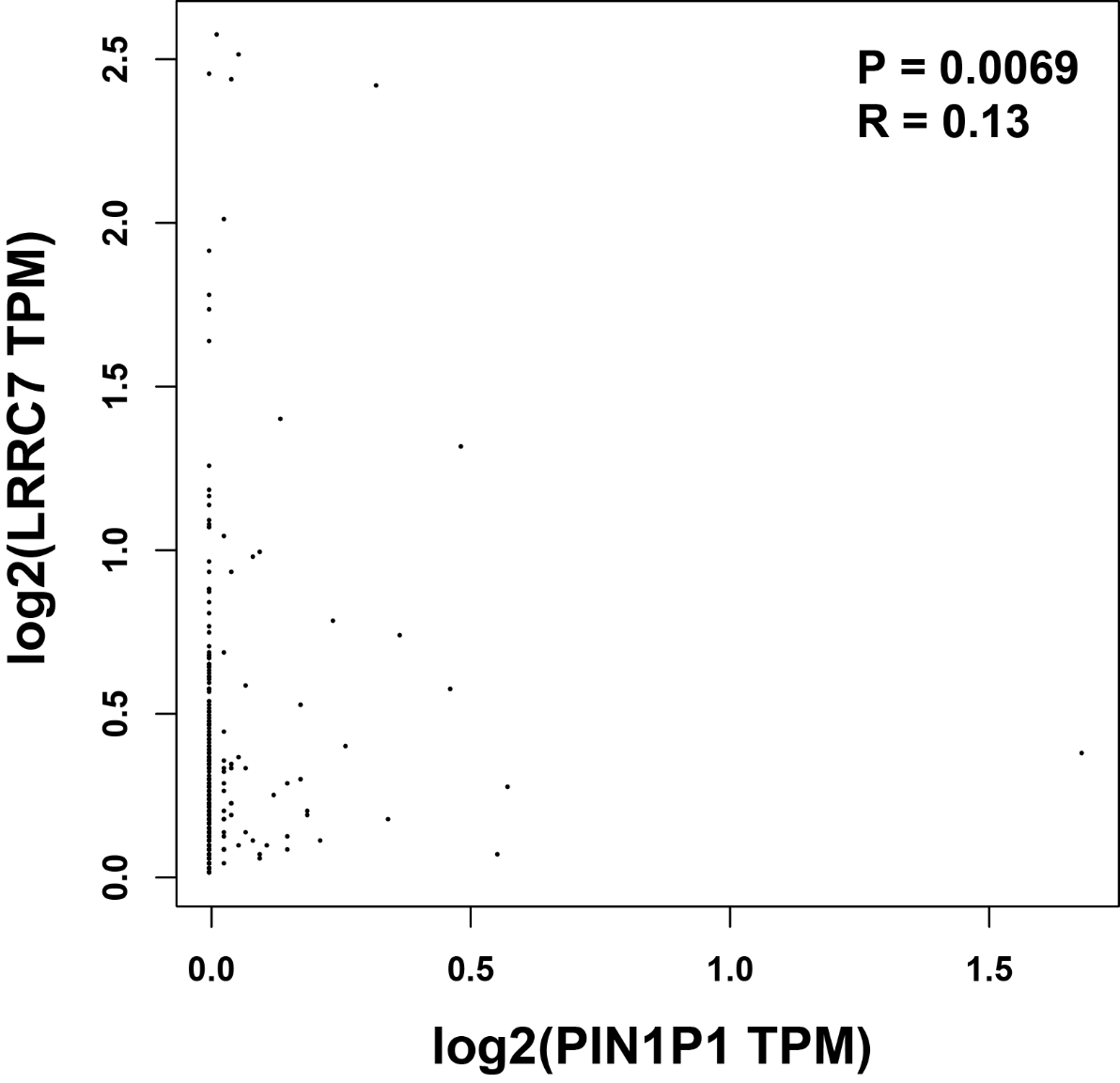
Supplementary Figure S2.** Expression of PIN1P1 and LRRC7 in gastric cancer samples based GEPIA database. PIN1P1 was weakly positively correlated with LRRC7 expression (R = 0.13, P = 0.0069).


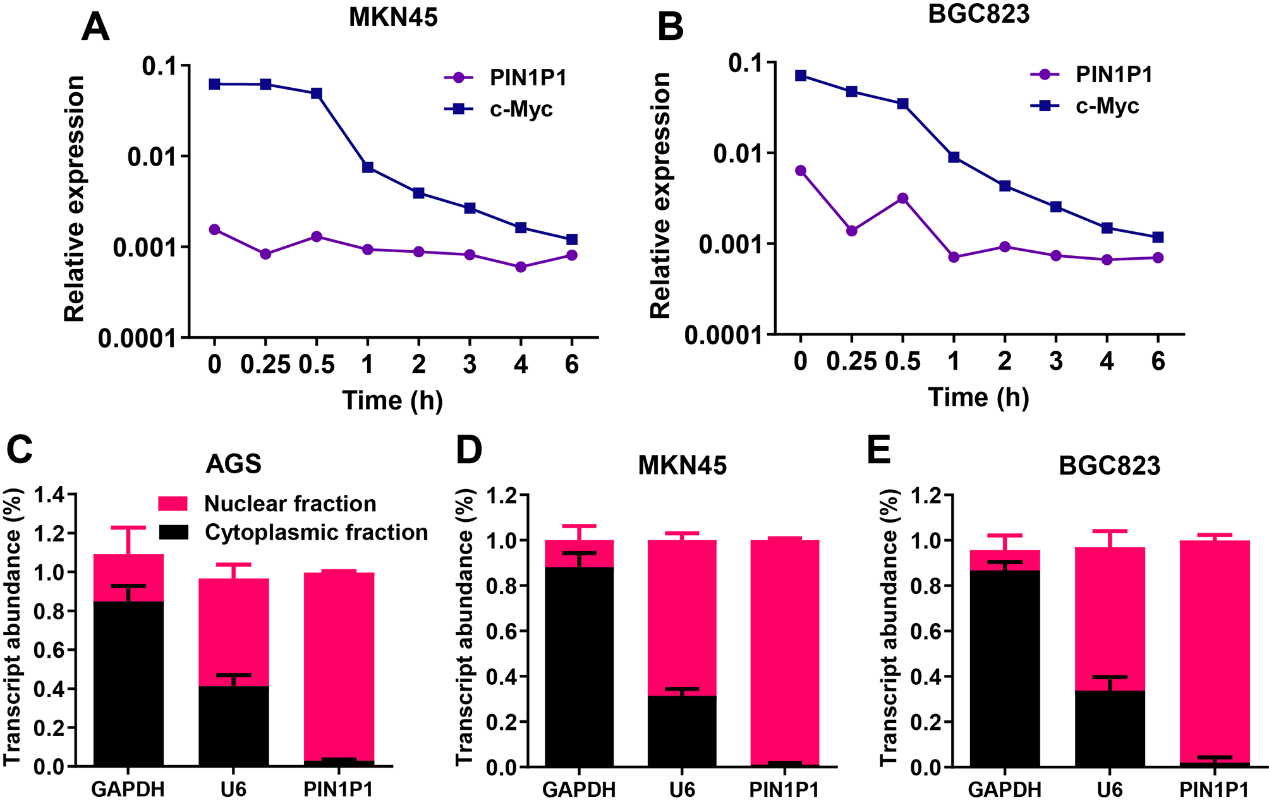


**Supplementary Figure S3. Stability and subcellular location of PIN1P1.**

(A–B) Actinomycin D chase assay showed that PIN1P1 had higher stability than c-Myc mRNA (control).

(C–E) Nuclear/cytoplasm fractionation was utilized to detect the subcellular location of PIN1P1 in gastric cancer cells, with U6 as a control for the nucleus and 18S for the cytoplasm.

**
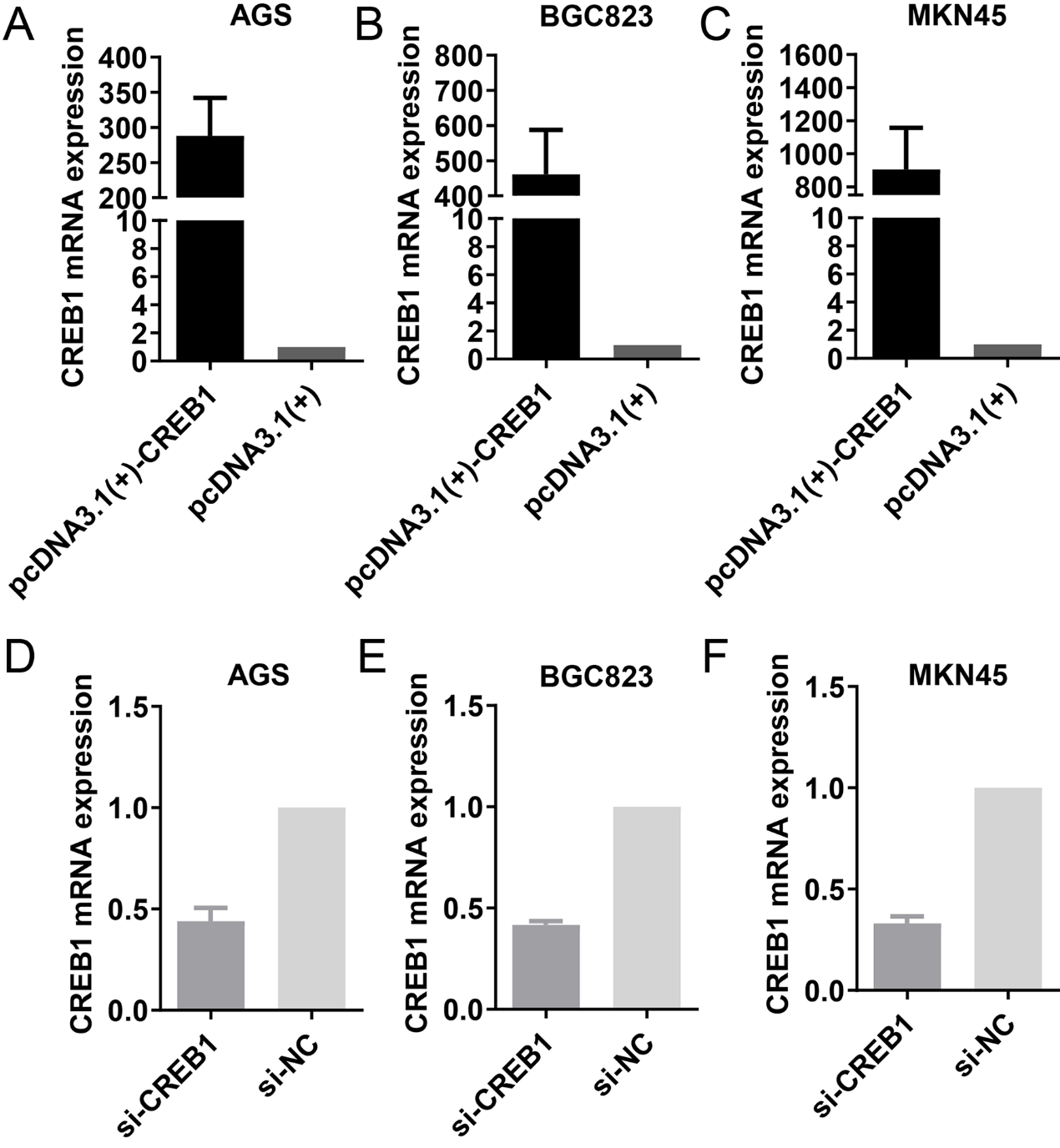
**

**Supplementary Figure S4. RT-qPCR validation of CREB1 expression in CREB1 overexpression plasmid (pcDNA3.1(+)-CREB1) or CREB1 siRNA (si-CREB1) transfected gastric cancer cells.** CREB1 overexpression plasmid or CREB1 siRNA could efficiently upregulate (A–C) or downregulate (D–F) the expression of CREB1, respectively.

**
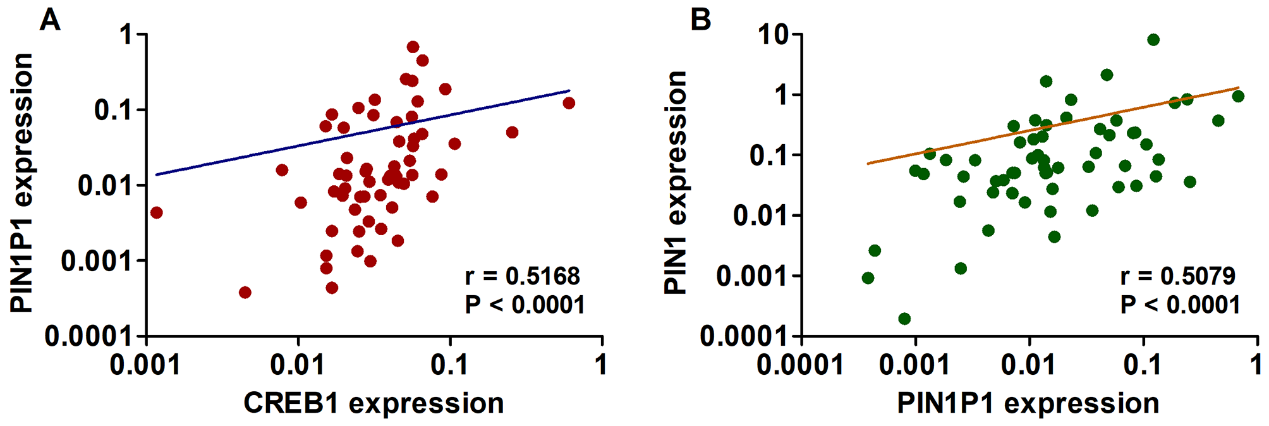
**

**Supplementary Figure S5.** Correlation analysis of CREB1, PIN1P1, and PIN1 expression in gastric cancer tissues.


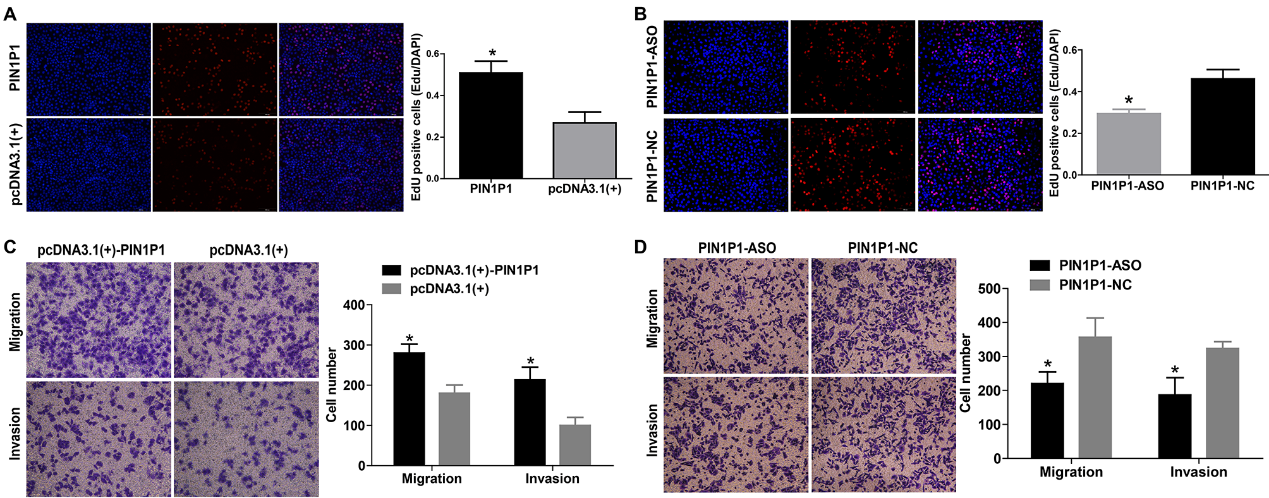


**Supplementary Figure S6. PIN1P1 enhanced gastric cancer MKN45 cells proliferation, migration, and invasion.**

(A–B) EdU assay revealed that PIN1P1 overexpression promoted cell proliferation.

(C–D) PIN1P1 promoted cell migration and invasion (magnification, × 200). Each treatment was three time replicated, and Mann Whitney test was used for comparison between two groups (^*^P < 0.05).


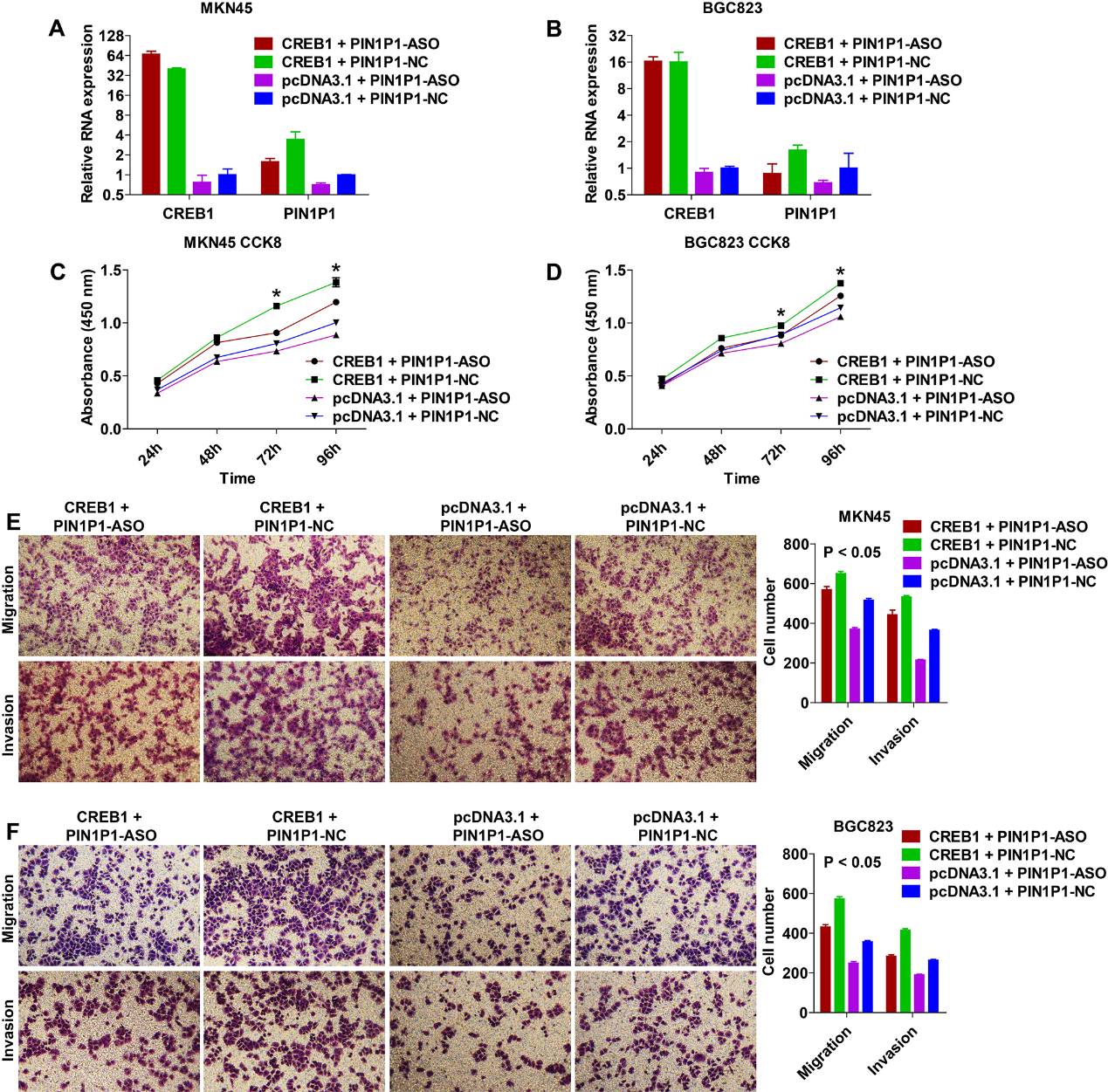


**Supplementary Figure S7. Silencing of PIN1P1 partially reversed CREB1-induced cell proliferation, migration, and invasion.**

(A–B) Expression of CREB1 and PIN1P1 mRNA expression in CREB1 and PIN1P1-ASO co-transfected gastric cancer cells.

(C–D) PIN1P1 inhibition partially reversed CREB1-induced cell proliferation, as determined by CCK8 assay.

(E–F) PIN1P1-ASO transfection in CREB1-expressing gastric cancer cells partially reversed CREB1-induced cell migration, and invasion. Each treatment was three time replicated, and Mann Whitney test was used for comparison between two groups (all P < 0.05).
